# Environmental Heterogeneity and Host Genotype Jointly Shape Endophytic Bacterial Community Composition Associated with an Endemic Chinese *Sphagnum* Species

**DOI:** 10.3390/microorganisms13112538

**Published:** 2025-11-05

**Authors:** Yan Liu, Xuechun Sun, Hongping Deng, Zhengwu Zhao

**Affiliations:** 1 College of Life Sciences, Chongqing Normal University, Chongqing 401331, China; 2 Chongqing Engineering Research Center of Specialty Crop Resources, Chongqing Normal University, Chongqing 401331, China; 3 School of Life Sciences, Southwest University, Chongqing 400715, China

**Keywords:** climate, host genotype, microbial diversity, peatland, *Sphagnum multifibrosum*

## Abstract

Peat mosses of the genus *Sphagnum* are keystone species in peatland ecosystems and play critical roles in carbon sequestration, nitrogen fixation, and hydrological regulation. Indeed, these ecological functions are largely mediated by endophytic bacteria associated with *Sphagnum*. Here, five populations of the endemic Chinese moss species, *S. multifibrosum*, were sampled across southern China in peatland (PH) and rock habitats (RH). High-throughput sequencing of 16S rRNA and nitrogenase (*nifH*) genes was applied to characterize overall endophytic bacterial diversity and diazotroph diversity associated with *S. multifibrosum*, respectively, alongside host microsatellite genotyping. Proteobacteria was the dominant endophytic bacterial phylum. The bacterial communities exhibited significant spatial separation between eastern and western communities and community dissimilarities significantly increased with increasing geographic distances. Environmental heterogeneity and host genetics jointly shaped endophytic bacterial community assemblage. Climate was the most important determinant influencing bacterial composition, followed by host genotype and habitat type. Temperature, precipitation, and nitrogen deposition were the primary environmental factors that influenced composition. Bacterial diversity and composition exhibited no statistically significant differences between the two habitats. Further, the richness and abundances of diazotrophs and methanotrophs from PH communities were higher than in RH communities. Co-occurrence network analysis suggested that RH bacterial networks had lower connectance but were more modularized and exhibited higher complexity than PH networks. These results highlight the ecological functions of peat mosses in carbon and nitrogen cycling and suggest a need to prioritize the conservation of *S. multifibrosum* in peatland environments under global climate change. The results also provide a framework to help future wetland management and biodiversity conservation efforts in China.

## 1. Introduction

Peatlands are the largest terrestrial carbon storage, holding up to one-third of the world’s soil carbon and serving as a natural carbon sink [1,2]. Peat mosses of the genus *Sphagnum* are keystone species and ecosystem engineers in peatlands [3,4] that play important roles in carbon sequestration, nitrogen fixation, and hydrological regulation [5]. However, these ecological functions are largely mediated by endophytic bacteria associated with *Sphagnum*. For example, Alphaproteobacteria and Cyanobacteria fix nitrogen [5,6], while methane-oxidizing bacteria mediate carbon cycling through methane conversion to CO_2_, subsequently providing substrates for photosynthesis [7,8,9]. These microbial activities thus supply essential nutrients for *Sphagnum* hosts, while also promoting carbon and nitrogen cycling in peatland ecosystems [10,11]. *Sphagnum*-associated endophytic bacteria are also beneficial to plant health [12], productivity [7,13,14], and adaptations to the extreme environmental conditions of peatlands [14,15,16].

Abiotic factors drive the assemblages of endophytic bacteria associated with peat mosses [3,17,18,19]. At a broad level, temperature profoundly impacts microbial composition and diversity [20,21,22], while nitrogen deposition can affect the community structure of nitrogen-fixing bacteria in peat mosses [23,24]. At the local level, water-table levels [9], pH [17,21,25], and nutrient availability [17,26,27] in peatlands drive community structure. In addition to abiotic influences, biotic factors like host genetics also play roles in structuring plant-associated microbiomes [28,29,30,31]. However, most studies of endophytic bacteria associated with peat mosses do not consider regional environmental gradients and generally neglect the effects of host genetics [19]. Consequently, the relative contributions of abiotic and biotic factors in shaping peat moss bacterial communities remain largely unknown.

Temperatures and nitrogen pollution are increasing under global climate change [32], leading to compositional shifts in endophytic bacteria associated with peat mosses [20,23,24,33]. In particular, reduced diversity of N_2_-fixing bacteria [20] and reduced relative abundances of diazotrophic methanotrophs [33] have been associated with warming. Similar patterns also emerge in peatland soils under warm and arid conditions, where both bacterial richness and α-diversity significantly decline [34]. Increased nitrogen deposition has also led to significant increases in the α-diversity of bacteria in *S. palustre* [24] and the abundances of soil bacteria in permafrost peatland [35]. Concomitantly, the abundances of Cyanobacteria inside *S. palustre* have significantly decreased [24]. These bacterial community changes could potentially alter the ecological functioning in peatlands [36,37,38]. Consequently, understanding the environmental determinants of endophytic bacterial assemblages in peat mosses will be helpful for predicting peatland responses to changing environments.

Comparative studies of endophytic bacterial communities across *Sphagnum* species [5,17,20,27] have revealed high host specificity [17,27]. Given the coexistence of multiple *Sphagnum* species within individual peatlands, comprehensive characterization of endophytic bacteria is essential for clarifying their functional contributions to peatland ecosystems. Although peatlands have attracted much scientific investigation as predominant habitats for *Sphagnum* species, large *Sphagnum*-dominated peatlands in southern China are rare due to temperature limitations. Most *Sphagnum* species are distributed sporadically on moist rocks in forests within areas of southern China at elevations exceeding 1000 m. Nevertheless, it remains unclear whether *Sphagnum* species from rock habitat (RH) exhibit similar endophytic bacterial communities compared to those from peatland habitat (PH), and especially bacterial functional groups related to carbon and nitrogen cycling.

Here, the endophytic bacterial communities associated with the endemic Chinese moss species, *S. multifibrosum*, were investigated. *S. multifibrosum* is a national second-class protected wild plant in China since 2021 that is found in southeast and southwest China [39] and is threatened due to resource exploitation [40]. Five populations from both PH and RH were sampled along a longitudinal gradient in southern China, covering the species’ geographic range. High-throughput sequencing of 16S rRNA and nitrogenase (*nifH*) genes was applied to characterize overall bacterial community diversity and diazotroph diversity, respectively, while host microsatellite (Simple Sequence Repeats, SSRs) genotyping was performed. The objectives were to (1) unveil the endophytic bacterial composition; (2) evaluate the relative importance of environmental heterogeneity and host genotype in shaping bacterial assemblages; and (3) compare diazotrophic and methanotrophic community composition between PH and RH. We hypothesized that environment and host genotype jointly shape endophytic bacterial community composition and PH communities exhibited higher richness and relative abundances of diazotrophs and methanotrophs than RH communities. The findings will advance our understanding of microbial community assembly in peat mosses and provide critical insights into conservation strategies for Chinese peatland ecosystems.

## 2. Materials and Methods

### 2.1. Sample Collection

**Figure 1:**
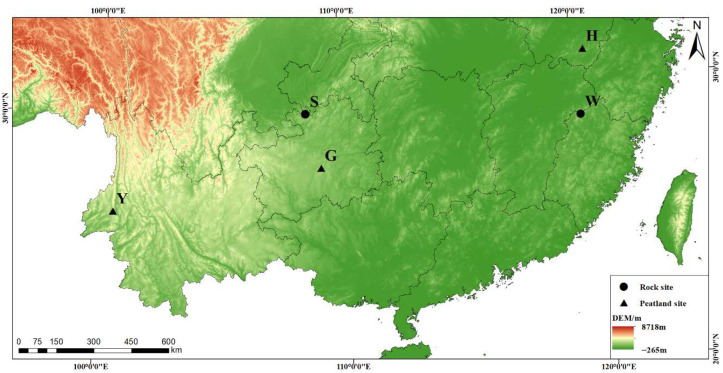
Sampling sites and habitat types of Chinese *Sphagnum multifibrosum*.

*S. multifibrosum* samples were collected from five sites in southern China during the summers 2017–2019 (Table S1), including at Huang Mt. in the Anhui province (H), Wuyi Mt. in the Fujian province (W), Longli Co. in the Guizhou province (G), Simian Mt. in the Chongqing municipality (S), and Tengchong city in the Yunnan province (Y) (Figure 1). Populations H and W were from southeastern China, while the others were from southwestern China. Sampling site longitudes ranged from 98°45′10″ to 118°9′28″ N, latitudes from 24°51′53″ to 30°8′31″ E, and altitudes between 1211 and 2147 m. The mean annual temperatures of the survey sites were 9.50–14.42 °C, and the mean annual precipitation levels were 1108–2119 mm during 1970–2000. Populations S and W were from forest rocks, while the others were from herbaceous dominated peatlands. RH samples were collected at 2 m intervals, while the four corners of peatlands were sampled. A total of 20 samples, including four replicates from each population (site) were ultimately collected and each sample contained 4–5 capitula (the apex of peat mosses). Samples were identified under a microscope according to Moss Flora of China [39].

### 2.2. Microsatellite Genotyping of S. multifibrosum

Genomic DNA was extracted from *S. multifibrosum* using a plant genomic DNA kit (TIANGEN, Beijing, China). A total of 13 microsatellite markers were selected for genotyping *S. multifibrosum*. The SSR loci number (i.e., 1, 4, 7, 9, 10, 14, 17, 18, 19, 20, 22, 29, and 30), primer sequences, microsatellite motifs, and fragment size ranges were provided in Table S2, as reported by Shaw et al. [41]. Multiplexing PCR methods also followed those of Shaw et al. [41]. SSR genotyping was performed with an ABI 3730XL sequencer (Applied Biosystems, Foster City, CA, USA) and the data were subsequently visualized and binned using the GeneMarker version 2.6 software (Softgenetics, State College, PA, USA).

### 2.3. DNA Extraction, PCR, and High-Throughput Sequencing of Endophytic Bacteria

Samples were first cleaned with sterilized water to remove surface attachments, then surface sterilized in 75% ethanol for 3 min followed by three washes with sterilized distilled water. The treated materials were then stored at −20 °C until subsequent DNA extraction. Endophytic bacterial community DNA was extracted using a soil DNA kit (Omega Bio-tek, Norcross, GA, USA). DNA was also extracted from the final rinse water as a disinfection control.

Nested PCR was conducted to amplify 16S rRNA gene, in which the first round PCR products were used as template for the second round. The outer primers were 799F (5′-AACMGGATTAGATACCCKG-3′) and 1492R (5′-GGTTACCTTGTTACGACTT-3′) that targeted the V5–V9 hypervariable 16S rRNA regions [42], while the inner primers were 926F (5′-AAACTYAAAKGAATTGACGG-3′) and 1392R (5′-ACGGGCGGTGTGTRC-3′) that targeted V6–V8 hypervariable regions [43]. To investigate the composition of diazotrophs associated with *S. multifibrosum*, *nifH* gene encoding the iron-containing dinitrogenase reductase subunit was amplified with the primers PolF (5′-TGCGAYCCSAARGCBGACTC-3′) and PolR (5′-ATSGCCATCATYRCCGGA-3′) [44]. PCR reactions contained 2 μL of DNA template, 1 μL of forward primer (10 μM), 1 μL of reverse primer (10 μM), 5 μL of 5× reaction buffer, 5 μL of 5× GC buffer, 2 μL of dNTPs (2.5 mM), 0.25 μL of Q5 High-Fidelity DNA Polymerase (New England Biolabs Inc., Ipswich, MA, USA), and ddH_2_O added to a final volume of 25 μL. The PCR program included the following steps: 98 °C for 2 min; 30 cycles of 98 °C for 15 s, 55 °C for 30 s, and 72 °C for 30 s; a final extension at 72 °C for 5 min. The amplicons were purified, quantified, pooled, and sequenced on the Illumina MiSeq PE250 platform (Illumina, San Diego, CA, USA) using standard protocols at Personal Biotechnology Co., Ltd. (Shanghai, China). The raw 16S rRNA and *nifH* gene sequences were deposited in the National Center for Biotechnology Information (NCBI) Sequence Read Archive (SRA) with the accession number PRJNA1327253.

### 2.4. Bioinformatic Analyses

The raw 16S rRNA and *nifH* gene sequencing data were processed with the QIIME2 version 2024.10 software [45]. The cutadapt plugin was first used to remove the primer sequences. The DADA2 plugin was then used for sequence quality control to merge paired reads, and remove chimeras. Reads were removed meeting either of the following criteria: (1) average Quality Score (*Q*) < 20; (2) presence of any ambiguous base (N); (3) paired reads with <10 bp overlap between the forward and reverse reads; and (4) presence of homopolymers with >8 bp. After quality filtering, the average sequence lengths were 463 bp for 16S rRNA gene and 317 bp for *nifH* gene. The filtered 16S rRNA and *nifH* gene sequences were clustered into operational taxonomic units (OTUs) at the 97% similarity threshold using the q2-vsearch plugin. Taxonomic classifications of sequences were assigned using the Naive Bayes classifier trained on the Greengenes2 database [46]. OTUs with relative abundances < 0.001% of the total reads across all samples [47] and assigned to “chloroplast” or “mitochondria” were removed. A total of 812,270 and 1,131,234 high-quality 16S rRNA and *nifH* gene sequences were obtained, ranging from 35,889–46,994 and 27,159–122,633 reads per sample, respectively (Table S3).

### 2.5. Environmental Variables

A total of 22 environmental variables, including 19 bioclimatic variables, solar radiation (SR), water vapor pressure (WVP), and nitrogen deposition (ND) were collected for analysis (Table S1). The bioclimatic variables, SR, and WVP were retrieved from the WorldClim database at a spatial resolution of 1 km [48], while ND data were obtained from National Ecosystem Science Data Center via the National Science & Technology Infrastructure of China [49].

### 2.6. Data Analyses

#### 2.6.1. Diversity Analysis and Indicator Identification

All data analyses were performed in R 4.4.2 [50]. Prior to diversity analyses, OTU data were rarefied to 32,300 sequences per sample for 16S rRNA gene and 24,443 sequences per sample for *nifH* gene, using the function rarefy_even_depth in “phyloseq” package (version 1.16.2; [51]). The rarefaction depth chosen is the 90% of the lowest sequencing. The Shannon diversity index of each sample (based on 16S rRNA and *nifH* genes, separately) was calculated using “vegan” package (version 2.62; [52]) based on OTU relative abundances. Values were then averaged for each population (sampling sites) and habitat type, respectively. Student’s *t*-tests were employed to evaluate statistical differences in Shannon diversity between PH and RH for 16S rRNA gene sequencing data, and Wilcoxon tests were applied for *nifH* gene. The choice of statistical methods depended on statistical assumptions for each dataset.

Bray–Curtis dissimilarities between bacterial communities were calculated using the function vegdist in “vegan” package (version 2.62; [52]) based on OTU relative abundances of 16S rRNA and *nifH* genes, and visualized using Principal Coordinates Analysis (PCoA) ordination with “ggplot2” package (version 3.5.2; [53]). Statistically significant differences in bacterial community composition between groups were assessed by Permutational Multivariate Analysis of Variance (PERMANOVA) with 999 permutations via the function adonis in “vegan” package (version 2.62; [52]). Statistically significant differences between groups along the first and second PCoA axes were further evaluated using the function multcompLetters4() of “multcompView” package (version 0.1-10; [54]). Linear discriminant analysis (LDA) Effect Size (LEfSe) analysis [55], as implemented in “microeco” package (version 1.15.0; [56]), was employed to identify significant bacterial indicators between segregated groups that were suggested by PCoA ordination, with LDA score threshold > 4.0. Geographic distances between samples were calculated in “geosphere” package (version 1.5-20; [57]) based on the geographic coordinates of each sampling sites. The linear relationships between community dissimilarities based on 16S rRNA and *nifH* genes and geographic distances were subsequently evaluated.

#### 2.6.2. Ordination Analysis

OTU abundance data were transformed by “hellinger” standardization. To avoid multicollinearity among the 22 collected environmental variables, Pearson correlation analysis was utilized with “stats” package for R 4.4.2 [50] to identify and eliminate highly correlated variables (|r| > 0.8). Five variables were retained and standardized using the Z-score method for subsequent analysis, including max temperature of the warmest month (BIO5), mean temperature of the warmest quarter (BIO10), precipitation of the wettest month (BIO13), ND, and SR. Environmental determinants of endophytic bacterial communities were detected, using the unimodal model Canonical Correspondence Analysis (CCA) based on the Detrended Correspondence Analysis (DCA) results. The significance of each environmental variable for the distribution patterns of bacterial communities was determined by PERMANOVA with 999 permutations, using the function envfit in “vegan” package (version 2.62; [52]).

Principal Component Analysis (PCA) was performed to reduce the dimensionality of the 22 collected environmental variables, with the first two principal components (PCs) retained as the “climate” factor, which explained ≥ 90% cumulative variance. Population structure analysis of *S. multifibrosum* was implemented in “LEA” package (version 3.2.0; [58]) based on SSR data. The ancestry proportions of each sample were used to represent host genotypes. The habitat factor was categorized into two types. Variance Partitioning Analysis (VPA) was carried out to quantify the relative contributions of climate, host genotype, and habitat to bacterial community composition based on 16S rRNA and *nifH* genes. The significance of each factor was tested using the function anova of “vegan” package (version 2.62; [52]). All ordination analyses, including DCA, CCA, PCA, and VPA, were conducted using “vegan” package (version 2.62; [52]).

#### 2.6.3. Co-Occurrence Network Analysis

Bacterial co-occurrence networks for 16S rRNA and *nifH* genes were constructed for each habitat of *S. multifibrosum*. Only OTUs that appeared in over two samples and ranked in the top 100 with highest relative abundance were selected. Spearman’s correlation coefficients (r) between the top OTUs at |r| ≥ 0.7 and false discovery rate (FDR) adjusted *p* < 0.01 were retained for network analysis. Co-occurrence networks were constructed using “igraph” package (version 2.2.1; [59]). The Gephi version 0.10.1 software [60] was employed to visualize the networks and calculate topological properties for each habitat.

## 3. Results

### 3.1. Bacterial Community Composition

**Figure 2:**
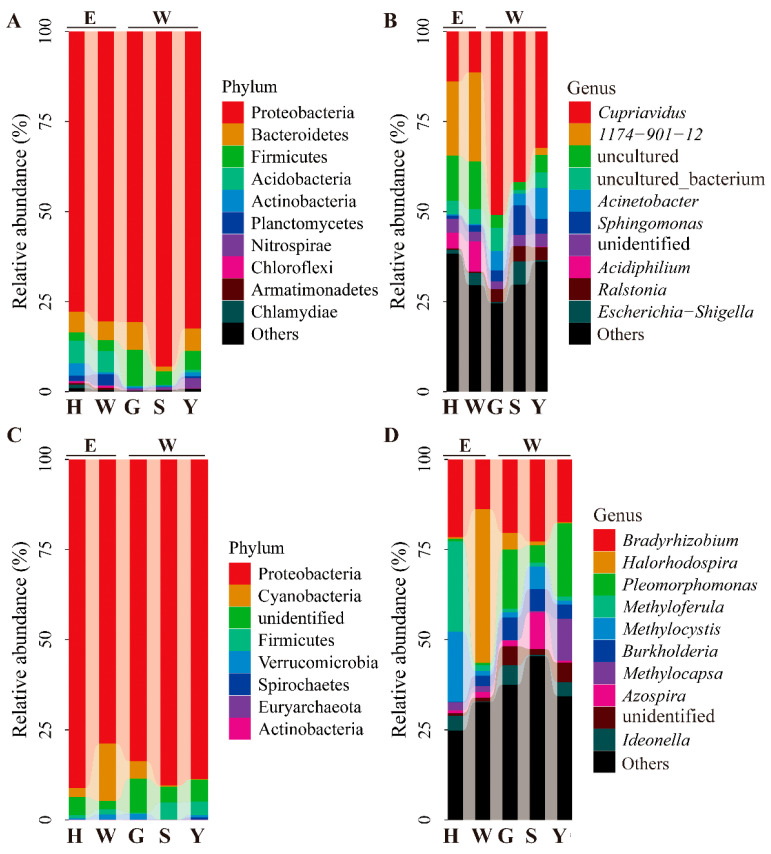
Bacterial community composition among five populations of *Sphagnum multifibrosum* considering 16S rRNA and *nifH* gene at the phylum (**A**,**C**) and genus (**B**,**D**) levels. E: eastern populations; W: western populations. H, Huang Mt. in the Anhui province; W, Wuyi Mt. in the Fujian province; G, Longli Co. in the Guizhou province; S, Simian Mt. in the Chongqing municipality; Y, Tengchong city in the Yunnan province.

16S rRNA gene OTUs were assigned to 29 phyla and 787 genera. The dominant phyla (relative abundances ≥ 1%) were Proteobacteria (82.90%), Bacteroidetes (5.24%), Firmicutes (4.89%), Acidobacteria (2.65%), Actinobacteria (1.18%), and Planctomycetes (1.02%, Figure 2A). The top 10 most abundant genera were *Cupriavidus* (30.01%), *1174-901-12* (9.50%), uncultured (7.29%), uncultured_bacterium (3.93%), *Acinetobacter* (3.60%), *Sphingomonas* (3.58%), unidentified (3.12%), *Acidiphilium* (2.56%), *Ralstonia* (2.46%), and *Escherichia*-*Shigella* (2.30%, Figure 2B). The *nifH* gene OTUs comprised 7 identified phyla and 109 genera, including the phyla Proteobacteria (86.54%), Cyanobacteria (4.75%), Firmicutes (2.20%), Verrucomicrobia (0.83%), Spirochaetes (0.14%), Euryarchaeota (0.03%), and Actinobacteria (0.01%, Figure 2C). The top 10 most abundant genera were *Bradyrhizobium* (19.19%), *Halorhodospira* (9.80%), *Pleomorphomonas* (8.60%), *Methyloferula* (5.99%), *Methylocystis* (5.92%), *Burkholderia* (3.81%), *Methylocapsa* (3.20%), *Azospira* (2.94%), unidentified genera (2.82%), and *Ideonella* (2.76%, Figure 2D). A total of 301 and 31 OTUs from 16S rRNA and *nifH* genes were shared among the five survey populations, respectively. The eastern population W and H harbored the greatest numbers of unique OTUs for the two genes, respectively (Figure S1A,B). Only 32 16S rRNA and 3 *nifH* OTUs were observed across all samples. Taxonomic core microbiome identified through occurrence data consistently included the phyla Proteobacteria and Firmicutes, along with the genera *Cupriavidus*, *Burkholderia*, and *Azospira*.

### 3.2. Distributions of Bacterial Communities and Indicator Taxa

**Figure 3:**
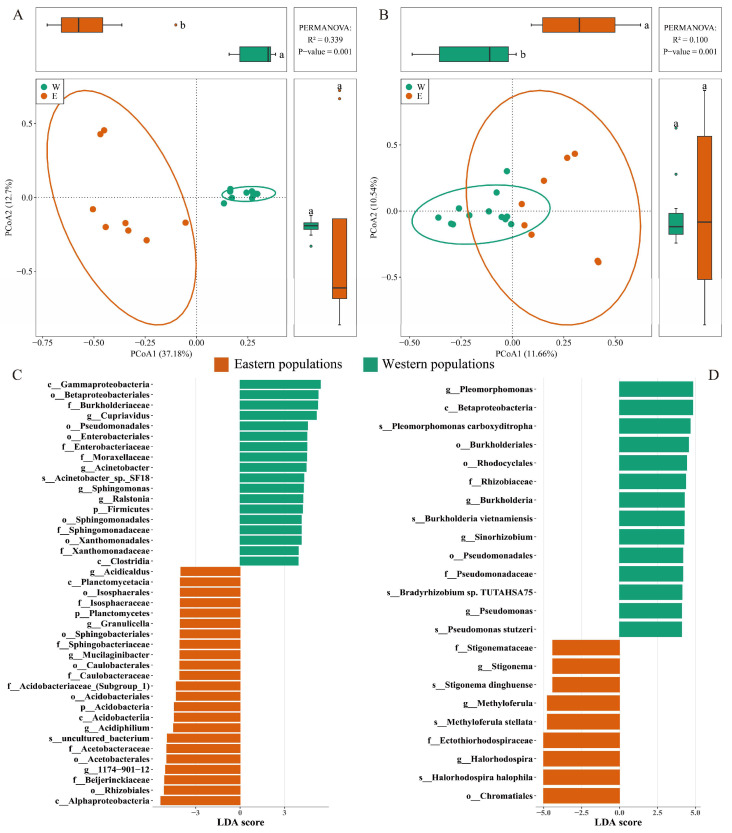
Variation in bacterial communities associated with *Sphagnum multifibrosum* based on Principal Coordinates Analysis (PCoA) of 16S rRNA (**A**) and *nifH* (**B**) genes. Indicator bacterial taxa in eastern and western populations of *S. multifibrosum* were identified based on Linear Discriminant Analysis (LDA) Effect Size (LEfSe) analysis of 16S rRNA (**C**) and *nifH* (**D**) genes. The bar chart shows bacterial taxa with *p* < 0.05 and LDA score > 4.0. “p__”, “c__”, “o__”, “f__”, “g__”, and “s__” represent phylum, class, order, family, genus, and species, respectively.

The 16S rRNA and *nifH* gene community composition exhibited significant spatial separation between eastern and western communities along PCoA1 (*p* < 0.01, Figure 3A,B) that may be a proxy for a longitudinal gradient. The dissimilarities in bacterial composition (y) based on 16S rRNA (y = 0.55325 + 0.00017x, R^2^ = 0.301, *p* < 0.001) and *nifH* genes (y = 0.89590 + 0.00005x, R^2^ = 0.208, *p* < 0.001) significantly increased with increasing geographic distances (x).

LEfSe analysis of 16S rRNA gene identified 23 indicator bacterial taxa in eastern populations and 18 in western populations (Figure 3C), while analysis of *nifH* gene revealed 9 and 14 significantly abundant taxa in the respective populations (Figure 3D). Considering the 16S rRNA gene, *1174-901-12* and *Cupriavidus* were the most enriched genera, with average relative abundances of 22.8% and 41.6% in eastern and western populations, respectively (Figure 3C). Considering the *nifH* gene, *Halorhodospira* and *Pleomorphomonas* were the most enriched genera, with average relative abundances of 21.6% and 13.9% in the respective populations (Figure 3D).

### 3.3. Abiotic and Biotic Effects on Bacterial Communities

**Figure 4:**
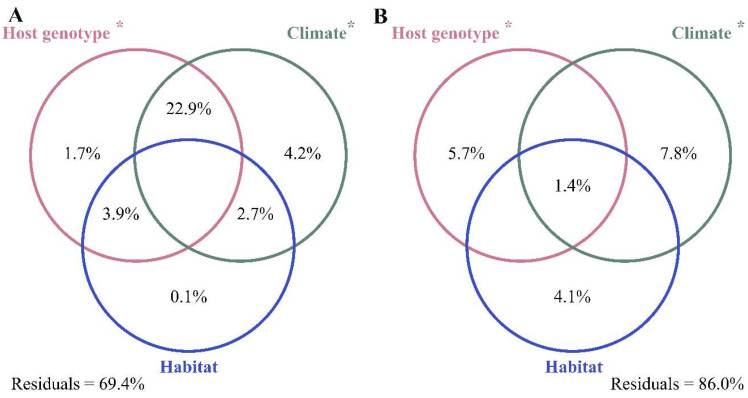
The effects of climate, host genotype, and habitat on bacterial communities associated with *Sphagnum multifibrosum* based on 16S rRNA (**A**) and *nifH* (**B**) genes. *: *p* < 0.05.

VPA revealed that climate, host genotype, and habitat type jointly explained 30.6% and 14.0% of variation in 16S rRNA (Figure 4A) and *nifH* (Figure 4B) genes, respectively. Notably, climate and host genotype significantly influenced bacterial community composition (*p* < 0.05). Indeed, climate was the most important determinant with the highest individual effect on bacterial community composition, followed by host genotype and habitat (Figure 4). Among abiotic variables, BIO5, BIO13, BIO10, and ND were significantly associated with bacterial communities, while SR was not. The impact of ND on diazotroph communities was stronger than that for overall endophytic bacterial communities (Table 1).

### 3.4. Differences in Bacterial Communities Between Two Habitats

**Figure 5:**
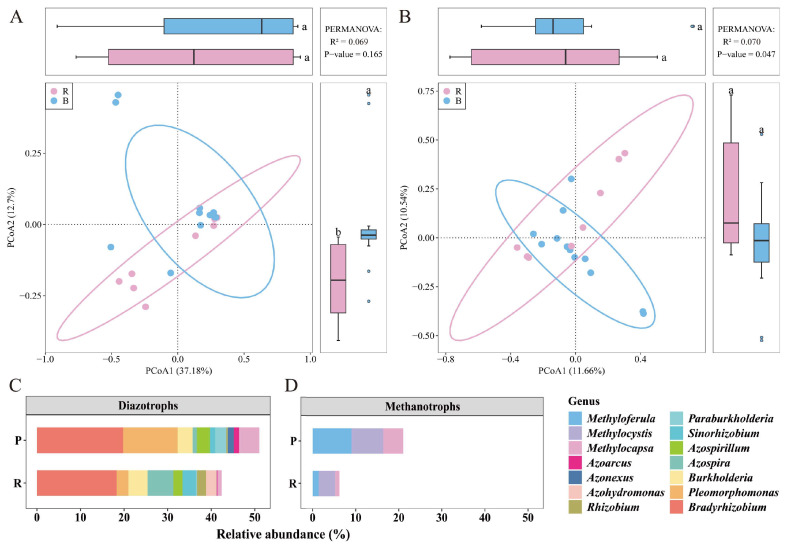
Variation in bacterial communities associated with *Sphagnum multifibrosum* from rock (R) and peatland (P) habitats based on Principal Coordinates Analysis (PCoA) of 16S rRNA (**A**) and *nifH* (**B**) genes. Diazotroph (**C**) and methanotroph (**D**) community members at the genus level with average relative abundances > 1% were specifically compared between the two habitats.

The Shannon index values among the five populations ranged from 5.14 (G) to 6.41 (Y) for 16S rRNA gene and from 4.75 (S) to 5.28 (G) for *nifH* gene (Table 2). The average RH community Shannon index was higher than that of PH based on 16S rRNA gene, while the opposite trend was observed for *nifH* gene. Nevertheless, statistically significant differences in bacterial diversity and composition were not observed between the two habitats based on either the 16S rRNA (Figure 5A) or *nifH* (Figure 5B) gene datasets (*p* > 0.05).

The compositions and relative abundances of diazotrophs and methanotrophs varied between PH and RH samples (Figure 5C,D). Specifically, the relative abundances of most diazotrophic bacterial genera including *Bradyrhizobium*, *Pleomorphomonas*, *Azospirillum*, and *Azoarcus* were higher in PH communities than in RH communities (Figure 5C). Similar trends were observed for methane-oxidizing bacterial genera including *Methylocapsa*, *Methyloferula*, and *Methylocystis* (Figure 5D). In contrast, the relative abundances of the diazotrophic bacterial genera *Burkholderia*, *Azospira*, *Azohydromonas*, *Rhizobium*, and *Sinorhizobium* were lower in PH samples than in RH samples (Figure 5C).

### 3.5. Bacterial Co-Occurrence Networks

**Figure 6:**
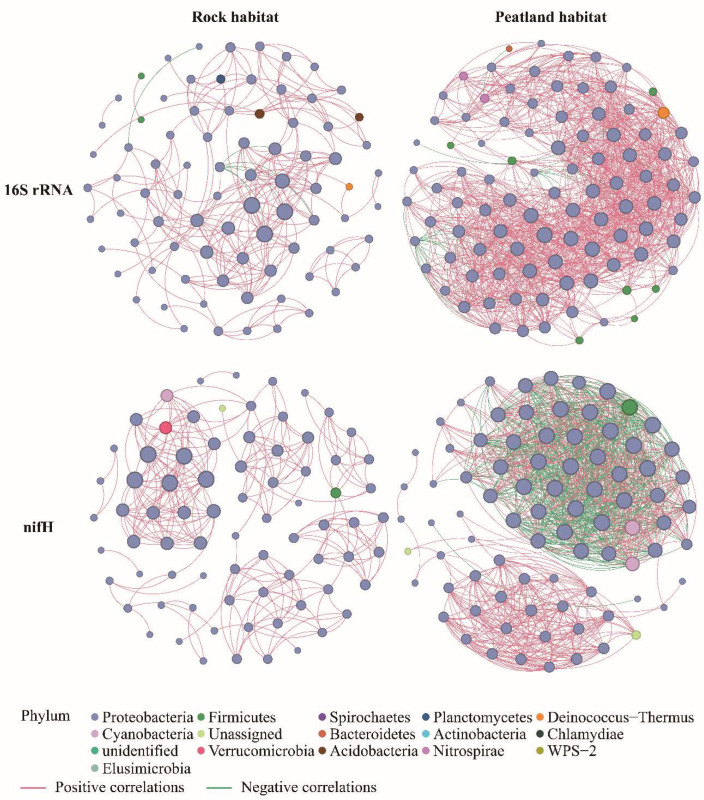
Co-occurrence networks based on 16S rRNA and *nifH* gene OTU distributions from rock and peatland habitats of *Sphagnum multifibrosum*. The size of each node is proportional to degree number and edge thickness is proportional to the absolute value of the correlation coefficient. Only phyla with relative abundances > 5% were analyzed in the networks.

Proteobacteria was the primary keystone taxa in bacterial co-occurrence networks (Figure 6). All networks exhibited distinct modular structures (with modularity values > 0.4). The numbers of edges, average degree, and graph density in PH networks were greater than in RH networks for both 16S rRNA and *nifH* genes, while the modularity and average clustering coefficients in RH networks were higher than in PH networks, indicating that the RH bacterial networks had lower connectance but were more modularized and exhibited higher complexity than PH networks. The ratio of negative to positive correlations in the 16S rRNA gene network was higher in the RH network, while the ratio was slightly higher in the *nifH* PH network (Table 3).

## 4. Discussion

This study characterized the endophytic bacterial communities associated with an endemic Chinese *Sphagnum* species and assessed the relative contributions of environmental heterogeneity and host genotype in shaping these communities at a regional scale. The results demonstrated that Proteobacteria dominated the core microbiome, while climate and host genotype significantly influenced bacterial community composition. Notably, PH communities exhibited higher richness and relative abundances of diazotrophs and methanotrophs compared to RH communities. These findings demonstrate the significant ecological functions of peat mosses in carbon and nitrogen cycling, emphasizing the conservation importance of peat mosses in peatland ecosystems.

### 4.1. Endophytic Bacterial Composition and Their Ecological Functions

Analysis of endophytic bacterial communities associated with *S. multifibrosum* across southern China revealed that the phylum Proteobacteria was the most abundant bacterial group and a core endophyte taxon, which are consistent with findings from other congeneric moss species [15,17,20,26,61] and non-*Sphagnum* mosses [62]. In addition, other abundant phyla were detected that contain N_2_-fixing organisms, including Bacteroidetes, Cyanobacteria, and Verrucomicrobia [63,64,65]. These phyla were among the most abundant, highlighting the importance of endophytic N_2_-fixation functionality in *S. multifibrosum*. Diazotrophs have been shown to fix up to 35% atmospheric nitrogen present in *Sphagnum* species biomass [10,11,12,66,67] and are the primary sources of nitrogen input in oligotrophic peatlands [68]. Consequently, carbon is sequestered in peatlands via these microorganisms.

Among the dominant bacterial phyla identified here, Proteobacteria, Bacteroidetes, Actinobacteria, and Firmicutes are widely distributed in peatlands [66]. Acidobacteria live in various types of peatlands [69,70] and participate in many biogeochemical cycles, including in carbon, nitrogen, sulfur, and iron cycling [71,72]. Verrucomicrobia are common in acidic geothermal environments [73] and bryophytes from moist and acidic environments [18,69]. Hence, these microorganisms may help *S. multifibrosum* adapt to the harsh environments of peatlands.

The most abundant genera identified by *nifH* gene analyses included *Bradyrhizobium* [35], *Pleomorphomonas* [74], and *Burkholderia* [7], which are capable to fix atmospheric nitrogen. All of the 10 most abundant genera identified in the *nifH* gene datasets (excluding an unidentified genus) belong to the phylum Proteobacteria, indicating the importance of N_2_-fixation by non-Cyanobacteria in *Sphagnum* species [61,75]. Some of the dominant genera included *Methyloferula* [76], *Methylocystis* [77], and *Methylocapsa* [78], which are well known methanotrophs that play important roles in *Sphagnum*-dominated peatlands [3]. Indeed, methanotrophs may provide up to 1/3 of the carbon required by peat mosses [8,9,10], consume peatland methane, and prevent methane release into the atmosphere [79]. Moreover, *Methyloferula*, accounted for ~25% of the transcribed *nifH* gene [3], has previously been considered the main source of bioavailable nitrogen and key bacterium that links N_2_-fixation with methane oxidation [26,79].

This study is the first to report that the genus *1174-901-12* belonging to the Beijerinckiaceae family is one of the most abundant endophytic bacterial genera associated with *Sphagnum* species (Figure 2B and Figure 3C). The prevalence of this genus may be related to the unique morphological characteristics of the moss host [19]. *S. multifibrosum* morphologically resembles the widely distributed *S. palustre* but has longer stem leaves with multiple fibrils in hyaline cells [39], where microorganisms can colonize [5]. The genus *1174-901-12* has also been found in beech and spruce [80], bamboos [81], and atmospheric tillandsioids [82]. Some members of this genus are methanotrophs and can fix nitrogen [83]. Moreover, *1174-901-12* is suggested to be a potential antagonist of plant pathogenic fungi [84] that may also benefit peach tree growth and polysaccharides production [85].

### 4.2. Abiotic and Biotic Influences on Bacterial Community Composition

Some previous studies have shown that different populations of the same *Sphagnum* host shared similar microbial communities [17,62]. Here, distinct spatial segregation in the bacterial communities was observed between the eastern and western *S. multifibrosum* (Figure 3). Similar biogeographic patterns have been detected for *S. magellanicum* [23] and *Arabidopsis thaliana* [30]. These results consequently contribute to our understanding of host–endophyte relationships and the role of biogeography in structuring these associations.

Climate, host genotype, and habitat type were associated with variation in endophytic bacterial community composition, indicating that environmental heterogeneity and host genetics jointly shape plant-associated microbial communities [30,86]. The effect of climate on the bacterial community composition within *S. multifibrosum* was stronger than that of host genotype (Figure 4). This difference likely arises from abiotic factors dominating the demographic histories of plant hosts, potentially leading to decoupling of host genotype from endophytes [30,31,86]. Thus, the role of host genetics in structuring microbial composition are primarily subordinate to environmental factors [29,30]. Notably, the distinctive spatial segregation of bacterial community composition observed here corresponded to the population genetic structure of *S. multifibrosum* (Figure S2), suggesting that these endophytic bacterial communities may co-evolve with their plant hosts [86,87,88,89]. The co-variance of microbial communities with host genotypes has been observed for other plant groups like *Arabidopsis thaliana* [28,30], *Boechera stricta* [31], *Glycine soja* [90], and *Coffea canephora* [91]. Nevertheless, disentangling the complex mechanisms underlying how genetic variation in *Sphagnum* hosts recruit and filter their endophytic bacteria under different environmental conditions requires further investigation and may not be addressable with the limited sample set of this study.

The upper temperature limit (BIO5 and BIO10), upper precipitation limit (BIO13), and ND significantly structured bacterial communities associated with *S. multifibrosum* (Table 1). Among these, temperature was the most important factor. Warming has been shown to alter microbial community composition in peat mosses or peat [2,20,70] and reduce the microbial diversity of peatland ecosystems [33,34,70,92,93]. Diazotroph and methanotroph communities are particularly sensitive to increased temperatures [20,33,70,92,93]. Precipitation was another key factor influencing endophytic bacterial community composition associated with *S. multifibrosum*, which has been observed for other plant-associated microbiomes [94,95,96]. Precipitation directly regulates peatland water table depth. When water levels decline, desiccation caused by drought will trigger the cessation of methane oxidation in peat mosses [97]. Conversely, when water levels rise, peat mosses submerged in water will lead to reactivation of their associated methanotrophs and subsequent oxidation of methane [8,98,99]. The stronger influence of ND on diazotroph communities relative to overall endophytic bacterial communities is notable. ND has been shown to significantly alter endophytic bacterial community composition in *Sphagnum* species [23,24].

### 4.3. Bacterial Functions Differ Between RH and PH Communities: Conservation Perspectives

*S. multifibrosum*-associated bacterial communities from RH and PH samples comprised abundant phyla associated with N_2_-fixation, including Proteobacteria, Cyanobacteria, and Verrucomicrobia (Figure 2C). These N_2_-fixing bacterial groups are also dominant in some other epilithic moss endophyte communities [100]. The relative abundances of some diazotrophs from RH communities were higher than those from PH communities (Figure 5C), suggesting an importance of moss hosts in modulating bacterial N_2_-fixation, regardless of habitat type. However, the total abundances of both diazotrophs and methanotrophs were higher in PH than in RH communities (Figure 5C). These differences could be attributed to anoxic environments inherent to peatlands. The diazotrophs detected in *S. multifibrosum* endophytic bacterial communities include *Pleomorphomonas* and methanotrophs (i.e., Methylocystis, *Methylocapsa*, and *Methyloferula*) that are anaerobic, precisely match the anoxic conditions of peatlands [74,77,78,101].

The abundances of functional bacteria are positively correlated with their functions [19,20,27], suggesting that the strength of ecological functioning is related to increased relative abundances of functional taxa. Furthermore, the low oxygen concentrations in PH could favor bacterial N_2_-fixing activities [26,102]. Hence, the contributions of carbon and nitrogen cycling activities in *S. multifibrosum* PH communities may be greater than for epilithic populations, due to higher abundances of bacteria that are functional in those cycles. Co-occurrence network analysis suggested that the PH bacterial network was probably under higher stress and likely more vulnerable than the RH network due to global change (Table 3). These results suggest the necessity of prioritizing PH conservation when considering *Sphagnum* protection strategies. Nevertheless, the N_2_-fixing activities of *S. multifibrosum* endophytic bacteria were not directly measured here and some studies have suggested that the functional performance in N_2_-fixation or methane oxidation is not related to bacterial composition, owing to functional redundancy [103]. Consequently, further activity-based studies are needed in the future.

The carbon sequestration activities of peatlands will help mitigate global warming, owing to their cooling effects [104]. Moreover, methanotrophs within peat mosses help limit the emissions of greenhouse gases into the atmosphere by oxidizing methane [9,98]. However, peat moss habitats are experiencing drastic changes. Consistent warming leads to risks of decreased productivity of *Sphagnum* species [4,105] and even mortality [33]. Accordingly, *Sphagnum* distributions in the middle and low latitudes of the northern hemisphere have been predicted to contract under future climate scenarios [106]. Such changes will likely affect the ecological functioning of peat mosses in carbon sequestration and biological N_2_-fixation. Although the carbon balance responses of peatlands remain uncertain in future climate change, some studies have simulated likely shifts from carbon sink to carbon source, thereby resulting in further warming amplification [107,108,109]. The vulnerability and resilience of *Sphagnum*-dominated peatland ecosystems to global warming consequently underscores the necessity and urgency for their conservation.

## 5. Conclusions

Here, the endophytic bacterial diversity of *S. multifibrosum*, an endemic moss species of China, was evaluated by sampling two contrasting *S. multifibrosum* habitats at the regional scale. The results suggested that bacterial community composition may contribute to the adaptation of *S. multifibrosum* to peatland environments and highlighted the ecological functions of peat mosses in carbon and nitrogen cycling. A 16S rRNA gene analysis uncovered the presence of a novel and abundant bacterial genus *1174-901-12* that exhibits host specificity, suggesting potential importance in preserving moss host populations. Environmental heterogeneity and host genetics jointly shaped bacterial community structures. Temperature, precipitation, and ND were the primary abiotic determinants associated with moss endophytic bacterial community composition. Observed differences in the abundances of diazotrophs and methanotrophs in the two habitats alongside bacterial network analysis suggested the need to prioritize the conservation of *S. multifibrosum* in peatlands in the face of future global warming. These results provide a framework that can help guide future wetland management and biodiversity conservation efforts in China. In practice, the biomass, coverage, and distribution range size of *S. multifibrosum* are proposed as visual early-warning indicators of peatland functional shifts in response to global warming. Future research could conduct a long-term monitoring in PH or carry out simulated warming experiments to explore the dynamic changes in endophytic bacterial communities associated with peat mosses under climate change. The integration of metagenomic and metatranscriptomic approaches will provide deeper insights into the roles of microbial communities in shaping peatland ecosystem functions and the molecular mechanisms of plant–microbe interactions.

## Figures and Tables

**Table 1:** Abiotic determinants of bacterial community composition inferred from Canonical Correspondence Analysis (CCA).

Gene	Variable	CCA1	CCA2	r^2^	*p*
16S rRNA	BIO5	0.678	0.735	0.917	0.001 ***
	BIO13	−0.996	0.089	0.912	0.001 ***
	BIO10	0.721	0.693	0.559	0.003 **
	ND	−0.990	−0.143	0.418	0.015 *
	SR	−0.997	−0.075	0.035	0.750
*nifH*	BIO5	−0.372	−0.928	0.969	0.001 ***
	ND	0.950	0.313	0.683	0.001 ***
	BIO13	0.951	0.311	0.679	0.001 ***
	BIO10	−0.223	−0.975	0.557	0.004 **
	SR	−0.470	0.883	0.028	0.800

BIO5: max temperature of the warmest month, BIO10: mean temperature of the warmest quarter, BIO13: precipitation of the wettest month; ND: nitrogen deposition, SR: solar radiation. * *p* < 0.05, ** *p* < 0.01; *** *p* < 0.001.

**Table 2:** Shannon diversity of bacterial communities associated with the five sampled populations of *Sphagnum multifibrosum* from two habitats in China.

Habitat	Rock Habitat		Peatland Habitat		
Population	W	S	H	G	Y
16S rRNA gene	6.41	5.14	6.18	5.14	5.79
* nifH * gene	5.28	4.75	4.97	5.28	4.80

W, Wuyi Mt. in the Fujian province; S, Simian Mt. in the Chongqing municipality; H, Huang Mt. in the Anhui province; G, Longli Co. in the Guizhou province; Y, Tengchong city in the Yunnan province.

**Table 3:** Topological properties of bacterial co-occurrence networks in rock and peatland habitats of *Sphagnum multifibrosum*.

Metric	16S rRNA Gene		*nifH* Gene	
	Rock Habitat	Peatland Habitat	Rock Habitat	Peatland Habitat
Number of nodes	82	95	77	76
Number of edges	238	1184	242	305
Average degree	5.805	24.926	6.286	8.026
Graph density	0.072	0.265	0.083	0.107
Modularity	0.633	0.383	0.732	0.696
Average clustering coefficient	0.687	0.672	0.846	0.768
Average path length	4.800	2.137	2.644	3.663
Eigenvector centrality	0.840	0.526	0.842	0.825
Average betweenness centrality	100.939	54.453	14.130	43.842
Negative correlations (%)	2.940	2.280	0.000	0.980
Positive correlations (%)	97.060	97.720	100.000	99.020

## Data Availability

The original contributions presented in this study are included in the article/Supplementary Materials. Further inquiries can be directed to the corresponding author.

## Supplementary Materials

The following supporting information can be downloaded at https://www.mdpi.com/article/10.3390/microorganisms13112538/s1. Figure S1: Venn diagrams showing the shared OTUs of the 16S rRNA (A) and *nifH* (B) gene among populations of *Sphagnum multifibrosum*. Figure S2: Population genetic structure and individual admixture among five populations of *Sphagnum multifibrosum* in China. Each bar represents a sampled plant individual, where different colors indicate individuals that are admixed for different genotype groups. Table S1: Sample information and environmental factors at five sampling sites of *Sphagnum multifibrosum* in China. Table S2: Microsatellite primer sequences, motifs, and fragment size ranges. Table S3: High-quality 16S rRNA and *nifH* gene sequences of each sample.
