# Supplementary material for: Environmental Heterogeneity and Host Genotype Jointly Shape Endophytic Bacterial Community Composition Associated with an Endemic Chinese Sphagnum Species
Source: Microorganisms. 2025 Nov 5;13(11):2538. doi: 10.3390/microorganisms13112538 (PMC12654153; doi:10.3390/microorganisms13112538)
Supplement: Supplementary file 1 [file microorganisms-13-02538-s001.zip › microorganisms-3962129-supplementary.pdf]

Table S1 Sample information and environmental factors at five sampling sites of *Sphagnum multifibrosum* in China.

Item	H	W	G	S	Y
Longitude (°)	118.15804	117.73209	107.04060	106.44379	98.75300
Latitude (°)	30.14215	27.83356	26.57660	28.51760	24.86500
Altitude (m)	1579	1361	1593	1211	2147
Collection date (yyyy-mm)	2018-06	2018-06	2017-07	2019-06	2017-08
SR ($kJ \cdot m^{-2} \cdot day^{-1}$)	14309.92	14306.50	12661.50	11984.17	15940.00
WVP (kPa)	1.30	1.35	1.32	1.25	1.25
ND ($kg \cdot ha^{-1} \cdot year^{-1}$)	15.79	13.47	12.24	14.48	7.06
BIO1 (°C)	9.50	12.20	12.93	12.32	14.42
BIO2 (°C)	6.18	7.38	6.87	5.56	9.58
BIO3	22.96	28.73	26.41	21.46	42.59
BIO4	748.82	668.84	683.06	731.35	462.33
BIO5 (°C)	22.90	25.10	25.30	25.00	25.10
BIO6 (°C)	-4.00	-0.60	-0.70	-0.90	2.60
BIO7 (°C)	26.90	25.70	26.00	25.90	22.50
BIO8 (°C)	16.62	15.65	19.52	19.28	19.28

BIO9 (°C)	1.92	5.47	4.12	2.92	8.13
BIO10 (°C)	18.45	20.15	21.00	21.05	19.28
BIO11 (°C)	0.00	3.68	4.12	2.92	8.13
BIO12 (mm)	2119.00	2048.00	1162.00	1108.00	1250.00
BIO13 (mm)	350.00	347.00	213.00	180.00	233.00
BIO14 (mm)	48.00	47.00	20.00	18.00	16.00
BIO15 (mm)	54.53	57.43	68.37	65.85	76.63
BIO16 (mm)	893.00	925.00	558.00	499.00	646.00
BIO17 (mm)	191.00	185.00	66.00	58.00	64.00
BIO18 (mm)	873.00	706.00	522.00	486.00	646.00
BIO19 (mm)	215.00	235.00	66.00	58.00	64.00

H, Huang Mt. in the Anhui province; W, Wuyi Mt. in the Fujian province; G, Longli Co. in the Guizhou province; S, Simian Mt. in the Chongqing municipality; Y, Tengchong city in the Yunnan province; SR, solar radiation; WVP, water vapor pressure; ND, nitrogen deposition; BIO1, annual mean temperature; BIO2, mean diurnal range; BIO3, isothermality; BIO4, temperature seasonality; BIO5, max temperature of the warmest month; BIO6, min temperature of the coldest month; BIO7, temperature annual range; BIO8, mean temperature of the wettest quarter; BIO9, mean temperature of the driest quarter; BIO10, mean temperature of the warmest quarter; BIO11, mean temperature of

the coldest quarter; BIO12, annual precipitation; BIO13, precipitation of the wettest month; BIO14, precipitation of the driest month; BIO15, precipitation seasonality; BIO16, precipitation of the wettest quarter; BIO17, precipitation of the driest quarter; BIO18, precipitation of the warmest quarter; BIO19, precipitation of the coldest quarter.

Table S2 Microsatellite primer sequences, motifs, and fragment size ranges.

Locus	Forward primer (label)	Reverse primer	Motif	Forward flank (bp)	Reverse flank (bp)	Reference
1	AACCACAAGTGAGCATTACC (FAM)	ACCTCTCCTCTCTGATTTCTG	CA	124	112	
4	TTGTGAGGAAGTGGTGTTG (HEX)	TCAGCAAGAGATTTGTGACC	CA	140	23	
7	TCCAATGACGGTAGGAAAC (HEX)	TCCAAGTGTCTTACAATGTCTG	GA	30	128	
9	GCATTTGATTACGAACAAGAG (FAM)	CGGATGAGCAGAAACAAC	CT	54	98	
10	GGGTTAGGGGATGATCCTG (FAM)	CTTCAGCCACGAATCCATT	GA	164	34	
14	TCCCCCACTCCTCTACTTG (HEX)	CTTGGATTCCTTTGCTTCTG	AG	68	109	
17	CTTCCCCCTTGAAACAAAC (FAM)	TGGGTGCTCTCCAGAATAG	AAG	95	43	Shaw et al. (2008)
18	CTCCTATTGGCGACAGATTTC (FAM)	CCTCGTTCTCTTCCTTCCAC	AAG	60	48	
19	GCAAACCCTAAAACACAGTG (FAM)	ATCGGCGTATCTTGATGTC	AAG	170	72	
20	ACCCAACGGACTCTACGG (FAM)	AACGCTGAAACAGACCTCG	TTC	119	145	
22	TCCCCAACACAAACCTTC (FAM)	GCTTTGAAGAAAGTTCCAGTG	GAT	32	34	
29	CTCATCAGCCCAGTCAGTCA (HEX)	ACCCACGCATCAAAAGAAAC	AAG	134	41	
30	ACCACCCTCCTCTCAATCCT (FAM)	AGTGTTTGCCAGTGCCTCTT	GAT	88	29	

Table S3 High-quality 16S rRNA and *nifH* gene sequences of each sample

Sample	16S rRNA gene	*nifH* gene
H1	41,537	95,602
H2	41,272	54,741
H3	39,763	122,633
H4	42,448	120,642
W1	40,587	40,347
W2	37,973	53,295
W3	41,180	54,209
W4	38,471	41,709
S1	41,617	27,159
S2	44,528	49,871
S3	40,471	50,221
S4	41,131	33,920
G1	35,889	41,301
G2	41,059	46,098
G3	40,924	48,541
G4	41,178	36,305
Y1	46,994	66,055
Y2	37,386	41,735
Y3	36,392	45,050
Y4	41,470	61,800
Total	812,270	1,131,234

Figure S1 Venn diagrams showing the shared OTUs of the 16S rRNA (A) and *nifH* (B) gene among five populations of *Sphagnum multifibrosum*.

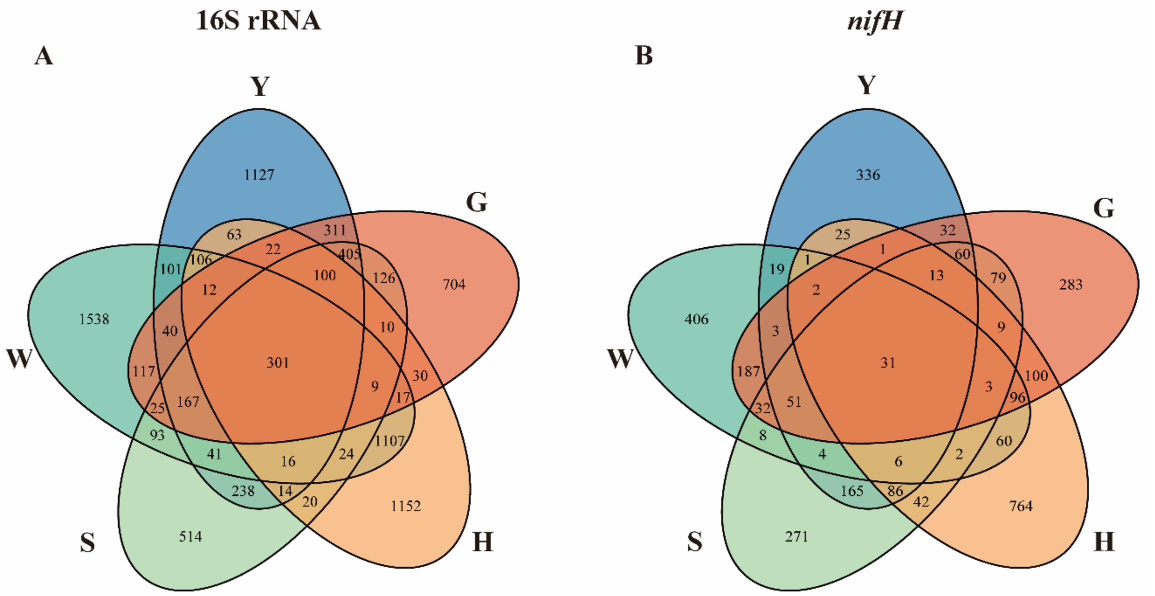

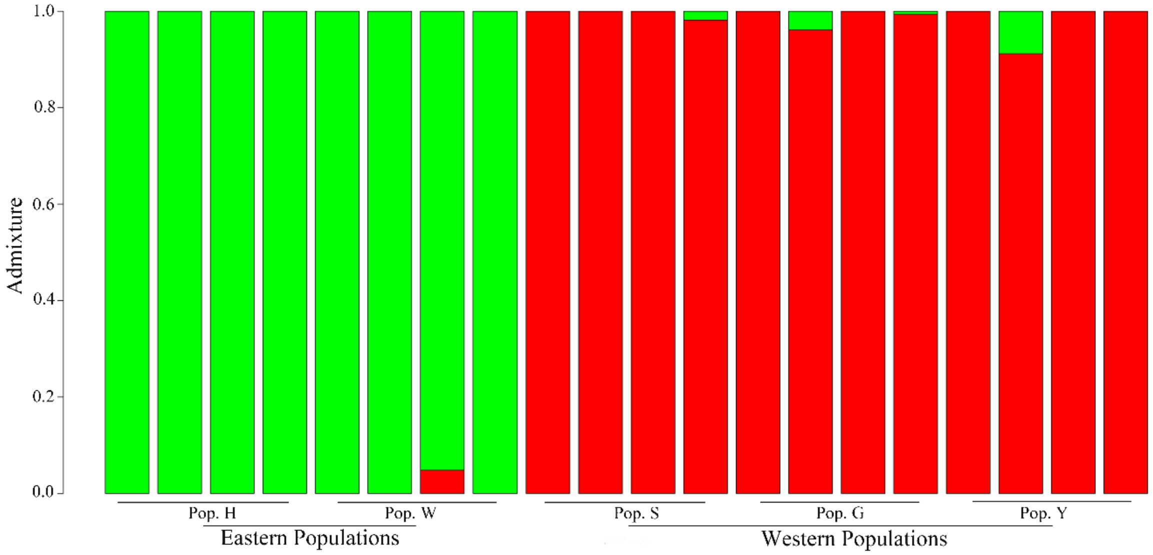

Figure S2 Population genetic structure and individual admixture among five populations of *Sphagnum multifibrosum* in China. Each bar represents a sampled plant individual, where different colors indicate individuals that are admixed for different genotype groups.
